# Validation of Bronchiectasis Health Questionnaire (BHQ) in Chinese Patients With Noncystic Fibrosis Bronchiectasis

**DOI:** 10.1155/carj/5551480

**Published:** 2026-07-22

**Authors:** Wang Chun Kwok, Terence Chi Chun Tam, Samuel Shung Kay Chan, James Chung Man Ho, Edmond Pui Hang Choi

**Affiliations:** ^1^ Department of Medicine, The University of Hong Kong, 102 Pokfulam Road, Pokfulam, Hong Kong Special Administrative Region, China, hku.hk; ^2^ School of Nursing, The University of Hong Kong, 5/F, Academic Building 3 Sassoon Road, Pokfulam, Hong Kong Special Administrative Region, China, hku.hk

**Keywords:** bronchiectasis, Bronchiectasis Health Questionnaire, Chinese, validation

## Abstract

**Background:**

The Bronchiectasis Health Questionnaire (BHQ) was developed to assess the health‐related quality of life (HRQOL) in patients with noncystic fibrosis (CF) bronchiectasis. This study aimed to translate, culturally adapt, and validate the BHQ for use among Chinese patients.

**Methods:**

The traditional Chinese version of the BHQ (TC‐BHQ) was created through a process of translation, cognitive debriefing interviews, and expert panel reviews. The psychometric evaluation of the TC‐BHQ encompassed assessments of factor structure, convergent validity, known‐group comparison, internal consistency, and test–retest reliability.

**Results:**

We recruited 150 non‐CF bronchiectasis patients. Confirmatory factor analysis (CFA) supported the one‐factor structure of the BHQ. In terms of concurrent validity, the TC‐BHQ total score showed a significant correlation with the St. George’s Respiratory Questionnaire (SGRQ) total score (*r* = −0.77,  *p* < 0.01). In addition, the TC‐BHQ total score significantly correlated with all SGRQ subscale scores, including the symptom score (*r* = −0.62,  *p* < 0.01), activity score (*r* = −0.60,  *p* < 0.01), and impact score (*r* = −0.75,  *p* < 0.01). There was a statistically significant change in the total score between baseline and follow‐up (*p* = 0.007). Both internal consistency and test–retest reliability of the TC‐BHQ were acceptable.

**Conclusion:**

TC‐BHQ is a promising tool for assessing HRQOL in patients with non‐CF bronchiectasis. It has the potential to be a useful instrument in both clinical and research settings.

**Summary**
• What is already known on this topic?
◦ The TC‐BHQ showed good correlation with the SGRQ.
• New knowledge added by this study?
◦ We demonstrate acceptable internal consistency and test–retest reliability of the TC‐BHQ.
• Implications for clinical practice or policy?
◦ TC‐BHQ has the potential to be a useful instrument in both clinical and research settings.

## 1. Introduction

Noncystic fibrosis (CF) bronchiectasis accounts for practically all cases that are seen commonly in Hong Kong and many other Chinese populations. Although there is no official epidemiological report on the prevalence of bronchiectasis in Hong Kong, in a study on validating the diagnostic coding for bronchiectasis in an electronic health record system in Hong Kong, there were 19,617 patients who had the diagnostic code of bronchiectasis among all public hospitals in Hong Kong from 2011 to 2020 [1].

Bronchiectasis is characterized by having microorganism colonization [2] as well as exacerbation. Exacerbation is one of the consequences of bronchiectasis, which is associated with a negative impact on morbidity, mortality, health‐related quality of life (HRQOL), and healthcare costs [3–11]. EMBARC Bronchiectasis Registry demonstrated that approximately 50% of patients had two or more exacerbations annually and one in three required hospitalizations [12]. The prognosis for bronchiectasis can be assessed by the FACED score [13]. The scoring system proposed risk factors for hospitalization, mortality, and severity of bronchiectasis.

In addition to the high prevalence and economic burden, assessment of HRQOL is important in chronic diseases such as bronchiectasis, as it can evaluate the overall impact on health from the patient’s point of view. HRQOL is severely impaired in bronchiectasis [14]. The health status of bronchiectasis patients can be evaluated with validated questionnaires, such as the St. George’s Respiratory Questionnaire (SGRQ) and Leicester Cough Questionnaire [15, 16]. Quality of Life Questionnaire–Bronchiectasis (QOL‐B) was the first disease‐specific, patient‐reported outcome measure for patients with bronchiectasis, which consisted of 37 items on eight scales [17]. However, these questionnaires are not practical for general use in the management of bronchiectasis patients as they are too lengthy, and the scoring system is very complicated. In light of this, Spinou et al. [18] developed a shorter and more practical tool, termed the Bronchiectasis Health Questionnaire (BHQ).

BHQ comprises 10 questions to assess HRQOL related to bronchiectasis in individuals. The recall period is 2 weeks. The items are scored on a 7‐point scale (ranging from 1 to 7). The total score ranges from 10 to 70, with a lower score indicating a more negative impact on HRQOL. Despite the questionnaire’s simplicity, the authors concluded that the questionnaire works well in a different population from the development cohort. While a simplified Chinese version of BHQ is available [19], a traditional Chinese version, which is crucial for effectively reaching the diaspora in regions such as Hong Kong, Taiwan, Singapore, and other Southeast Asia, has not yet been developed. This absence limits the questionnaire’s applicability and utility within these communities, where traditional Chinese is predominantly used.

Given the significance of the health impact and disease burden of bronchiectasis in Hong Kong, we need a tool for assessing HRQOL of Chinese patients with bronchiectasis. As BHQ has been developed, it is crucial to translate it into traditional Chinese and properly validate it to be used in target populations.

## 2. Materials and Methods

### 2.1. Study Design

The study comprised two phases. The first phase focused on the assessment of the content validity of the traditional Chinese version of the BHQ (TC‐BHQ). The second phase was an observational study, which included a psychometric evaluation of the TC‐BHQ to assess its validity and reliability.

### 2.2. Setting

The study was conducted in Queen Mary Hospital (QMH), Hong Kong. QMH is one of the major regional hospitals in Hong Kong and a university‐affiliated tertiary respiratory referral center, with a designated bronchiectasis clinic for managing patients with non‐CF bronchiectasis of different disease severity.

### 2.3. Eligibility Criteria of Patients

The inclusion criteria included Chinese patients with non‐CF bronchiectasis aged at or above 18 years old who were able to understand and read traditional Chinese. The diagnosis of bronchiectasis was confirmed by high‐resolution computed tomography (HRCT) findings of bronchiectasis that were verified by radiologists. Exclusion criteria were patients who were unable to complete the survey due to hearing or cognitive impairment, or who could not speak Chinese.

#### 2.3.1. Phase 1: Assessment of the Content Validity

A research coordinator performed the first translation of the original version of the BHQ into the traditional Chinese language (TC‐BHQ), which was further edited by the author WCK. Cognitive debriefing interviews were conducted to evaluate the content validity of the TC‐BHQ. Three aspects were assessed: the scale’s clarity, relevance, and appropriateness [20].

A convenience sample of 14 Chinese patients with bronchiectasis (5 males and 9 females) was recruited from the clinic. Each patient completed the TC‐BHQ, followed by a semistructured interview that included five questions about their perception of the scale. The first three questions required patients to rate each item in the BHQ on a four‐point Likert scale in terms of clarity, relevance, and appropriateness. Ratings ranged from 1 (*not at all clear/relevant/appropriate*) to 4 (*completely clear/relevant/appropriate*). The final two questions asked patients to describe their understanding of each item and to suggest any rewording for parts they found unclear, irrelevant, or inappropriate [20].

The research team members reviewed the findings from the cognitive debriefing interviews, with the review identifying any discrepancies between the patients’ interpretations and the intended meaning of the translated TC‐BHQ. Patient‐recommended rewordings were considered, and question items were revised as necessary to enhance clarity, relevance, and appropriateness. For example, Items 4 and 6 were reworded in this process. In addition, two physicians (TCCT and SSK Chan) were invited to evaluate the content validity of the scale.

#### 2.3.2. Phase 2: Psychometric Evaluation

An observation survey study was carried out to evaluate the psychometric properties of the TC‐BHQ. 150 patients with non‐CF bronchiectasis, as confirmed by HRCT of the thorax, were recruited in this phase. 50 patients with non‐CF bronchiectasis were invited for follow‐up interviews after 4 weeks to assess the test–retest reliability of the scale. In addition, 65 patients with follow‐up data were included to evaluate the responsiveness of the scale. These 65 patients were followed in the bronchiectasis clinic with BHQ measured for another noninterventional study. The mean follow‐up duration for these 65 patients was 19.7 ± 7.5 months from the initial recruitment date.

### 2.4. Study Instruments

#### 2.4.1. BHQ

The instrument comprises 10 questions to assess HRQOL related to bronchiectasis in individuals. The recall period of the BHQ is 2 weeks. The items are scored on a 7‐point scale (ranging from 1 to 7). After score transformation, the total score ranges from 0 to 100, with a lower score indicating a more negative impact on HRQOL.

#### 2.4.2. SGRQ

The SGRQ is a disease‐specific instrument designed to measure the impact of obstructive airway disease on overall health, daily life, and perceived well‐being. It consists of two parts with 50 items. It addresses the frequency and severity of patients’ symptoms, as well as activities that cause or are limited by breathlessness. The scores range from 0 to 100, with higher scores indicating more limitations. The minimally important difference is a mean change of 4 units for slightly efficacious treatment, 8 units for moderately efficacious change, and 12 units for very efficacious treatment [21, 22]. The SGRQ has been validated in patients with bronchiectasis and is considered a valid and sensitive instrument for determining the quality of life in bronchiectasis patients [23].

#### 2.4.3. Demographic and Clinical Data

Demographic data (including age, sex, and smoking status), along with clinical data and investigations (such as the etiology of bronchiectasis, comorbidities, treatment records, spirometry results, sputum culture results, and FACED score), were collected at the recruitment visit.

### 2.5. Statistical Analysis

For the Phase 1 assessment of content validity of TC‐BHQ, content validity indices (CVIs) for each item’s clarity, relevance, and appropriateness were determined by the proportion of participants who gave the item a rating of 3 or 4 on a four‐point scale. The standard for good content validity was set as a CVI of 80% or above [20].

For psychometric evaluation, the following analyses were conducted [24]:

- Factor structure: Confirmatory factor analysis (CFA) was conducted to validate the factor structure of the TC‐BHQ. The model’s goodness‐of‐fit was evaluated using several metrics: standardized root mean square residual (SRMR), root mean square error of approximation (RMSEA), comparative fit index (CFI), and Tucker–Lewis index (TLI), following guidelines set by Hu and Bentler [25]. A model fit is deemed good if the SRMR is close to or below 0.08, the RMSEA is up to 0.06, and the CFI and TLI values exceed 0.9, with values above 0.90 being acceptable and those above 0.95 being considered excellent [25].
- Concurrent validity: Pearson’s correlation coefficients were determined to describe the relationship between TC‐BHQ and SGRQ scores.
- Known‐group validity: An independent *t*‐test will be used to compare the BHQ scores between patients with bronchiectasis of ≥ 3 or < 3 lobes involved. Cohen’s d effect size will also be calculated. The number of lobes involved reflects the extent of bronchiectasis, which was incorporated in various scoring systems such as the bronchiectasis severity index (BSI) and FACED score.
- Internal consistency: The internal consistency of TC‐BHQ was examined using Cronbach’s alpha. A Cronbach’s alpha of 0.7 or higher is indicative of acceptable internal consistency [26].
- Responsiveness: The responsiveness of the TC‐BHQ was evaluated using a paired *t*‐test to compare the mean total scores between baseline and follow‐up. Cohen’s *d* effect sizes were also calculated.
- Test–retest reliability: To assess the 4‐week test–retest reliability of the TC‐BHQ, an intraclass correlation coefficient (ICC) was utilized. An ICC of 0.7 or above is considered to denote good test–retest reliability [26].

### 2.6. Ethics

The study was approved by the Institutional Review Board of the University of Hong Kong and Hospital Authority Hong Kong West Cluster (approval numbers: UW 22‐656 and UW 22‐317). Written informed consent was obtained from each study participant. We obtained permission for copyright using the original version of the BHQ from Dr. Surinder Birring of King’s College Hospital, UK.

## 3. Results

### 3.1. Content Validity

Among male patients, the CVIs for clarity, relevance, and appropriateness were above 80% for all items. Among female patients, the CVIs for relevance and appropriateness were also above 80% for all items. However, the CVIs for clarity were above 80% for all items except for Item 4 (66.7%) and Item 6 (77.8%).

### 3.2. Baseline and Clinical Characteristics of the Psychometric Evaluation

In total, 150 patients with non‐CF bronchiectasis were recruited, comprising 108 females (72%) and 42 males (28%). The mean age was 70.5 years, with a standard deviation of 10.4 years. A subset of 66 patients (44%) had bronchiectasis involving fewer than 3 lobes, while the remaining 84 (56%) had involvement of ≥ 3 lobes. The results are summarized in Table 1.

**TABLE 1:** Baseline demographic and clinical characteristics.

Clinical characteristics	Whole cohort (*n* = 150)
Age (years), mean ± SD	70.5 ± 10.4
Gender	
Male	42 (28.0%)
Female	108 (72.0%)
Smoking status	
Current smoker	3 (2.0%)
Ex‐smoker	15 (10.0%)
Nonsmoker	132 (88.0%)
BMI (kg/m^2^), mean ± SD	22.3 ± 4.9
FEV_1_ (L), mean ± SD	1.75 ± 0.52
FEV_1_ (% predicted) mean ± SD	93.0 ± 20.5
FVC (L), mean ± SD	2.47 ± 0.70
FVC (% predicted) mean ± SD	98.4 ± 18.5
FEV_1_/FVC ratio (%), mean ± SD	70.9 ± 10.3
mMRC dyspnea scale	
0	46 (30.7%)
1	63 (42.0%)
2	31 (20.7%)
3	9 (6.0%)
4	1 (0.7%)
Extent of involvement ≥ 3 lobes	84 (56.0%)
*Pseudomonas aeruginosa* colonization	42 (28.0%)
FACED score, median [IQR]	2 [1–3]

*Note:* mL, milliliter; FEV_1_, forced expiratory volume in one second.

Abbreviation: BMI, body mass index; FVC, forced vital capacity; mMRC, Modified Medical Research Council; SD, standard deviation.

### 3.3. Validity

The CFA supported a one‐factor structure for the TC‐BHQ. The RMSEA was 0.042, and the SRMR was 0.081. The CFI stood at 0.96, with a TLI of 0.95.

Regarding concurrent validity, the TC‐BHQ total score correlated significantly with the SGRQ total score (*r* = −0.77,  *p* value < 0.01), indicating a strong relationship. Furthermore, the TC‐BHQ total score showed significant correlations with all the SGRQ subscale scores, including symptom score (*r* = −0.62,  *p* value < 0.01), activity score (*r* = −0.60,  *p* value  <  0.01), and impact score (*r* = −0.75,  *p* value < 0.01). Table 2 shows the results.

**TABLE 2:** Concurrent validity (*n* = 150).

TC‐BHQ total score		
SGRQ	Pearson’s correlation coefficients (95% CI)	*p* value
Symptom score	−0.62 (−0.71, −0.51)	< 0.01
Activity score	−0.60 (−0.70, −0.49)	< 0.01
Impact score	−0.75 (−0.81, −0.67)	< 0.01
Total score	−0.77 (−0.83, −0.70)	< 0.01

In the known‐group comparison, no statistically significant difference was found in the TC‐BHQ total scores between participants with bronchiectasis affecting fewer than 3 lobes versus those with 3 or more lobes involved. Table 3 shows the results.

**TABLE 3:** Know‐group comparison (*n* = 150).

	< 3 lobes (*n* = 66) Mean (SD)	3 or more lobes (*n* = 84) Mean (SD)	*p* value	Cohen’s *d* effect size
TC‐BHQ total score	64.55 (9.34)	66.59 (10.41)	0.216	0.20

Regarding the responsiveness of the TC‐BHQ, a statistically significant improvement in the total score was observed between baseline (mean: 65.80, standard deviation: 10.10) and follow‐up (mean: 70.64, standard deviation: 15.86), with a *p* value of 0.007 and a Cohen’s *d* effect size of 0.35.

### 3.4. Reliability

The TC‐BHQ yielded a Cronbach’s alpha coefficient of 0.68. Item analysis showed that Item 4 (“My chest has felt clear”) demonstrated poor correlation with the total score, with a corrected item‐to‐total correlation of 0.09. Table 4 shows the result. For test–retest reliability, 50 participants completed the follow‐up questionnaire 4 weeks postbaseline, resulting in an ICC of 0.73 for TC‐BHQ. The results are illustrated in Table 5 **.**

**TABLE 4:** Internal consistency (*n* = 148).

	Corrected item‐total correlation	Cronbach’s alpha if item deleted
Item 1	0.48	0.63
Item 2	0.45	0.63
Item 3	0.41	0.65
Item 4	0.09	0.71
Item 5	0.35	0.66
Item 7	0.53	0.62
Item 6	0.38	0.65
Item 8	0.25	0.68
Item 9	0.31	0.67
Item 10	0.35	0.66

*Note:* Two participants were excluded from the analysis due to missing values.

**TABLE 5:** Test–retest reliability (*n* = 50).

	Baseline Mean (SD)	Follow‐up Mean (SD)	*p* value	ICC
TC‐BHQ total score	68.65 (9.86)	69.87 (9.46)	0.227	0.73 (0.57, 0.84)

## 4. Discussion

The present study sought to evaluate the psychometric properties of the TC‐BHQ in non‐CF bronchiectasis patients. Our findings offer insights into the TC‐BHQ’s reliability and validity as a measure for assessing HRQOL in this patient population. The translation and validation of BHQ into a traditional Chinese version as TC‐BHQ allows clinicians and researchers to properly evaluate the HRQOL of patients with non‐CF bronchiectasis, which is a disease commonly seen among this ethnic group.

While symptoms, lung function, and exacerbations are considered to be the key clinical and laboratory parameters in the management of chronic respiratory diseases, the HRQOL of patients is also another important aspect that should not be forgotten. In the past, SGRQ had been used as one of the gold standards in assessing HRQOL of patients with chronic respiratory diseases; it also has the disadvantage of being lengthy, time‐consuming to complete, and not disease‐specific. QOL‐B also has the disadvantage that it is too lengthy to be used in usual clinical settings. While in other respiratory diseases, different simpler versions of disease‐specific HRQOL assessment tools, such as mini‐AQLQ [27] in asthma, have been developed back in 1999. A simple and easy‐to‐use disease‐specific HRQOL assessment tool is lacking in bronchiectasis, until Spinou et al. [18] developed BHQ in 2017, which is a brief, valid, and repeatable, self‐completed health status questionnaire for bronchiectasis. However, to allow its use in different languages, translation and subsequent validation are needed, which have been performed in other languages such as Danish, Korean [28], and Portuguese [29]. In view of the high prevalence of non‐CF bronchiectasis in Hong Kong and other countries and places with a traditional Chinese‐speaking population, the translation and validation of BHQ into TC‐BHQ are crucial.

The factor analysis supported the one‐factor structure of the TC‐BHQ, with RMSEA, SRMR, CFI, and TLI indices all within acceptable to excellent ranges, indicating a good fit of the model. This suggests that the TC‐BHQ is a structurally sound instrument for capturing the HRQOL in bronchiectasis patients, consistent with the theoretical underpinning of the questionnaire design.

The strong correlation between the total TC‐BHQ and SGRQ scores underscores the TC‐BHQ’s concurrent validity, demonstrating its capacity to measure a construct similar to that of an established instrument, the SGRQ. Our study did not find a statistically significant difference in TC‐BHQ scores between patients with bronchiectasis involving fewer than three lobes versus those with three or more lobes. It could be explained by the fact that the radiological extent of bronchiectasis is the only parameter to assess the severity of bronchiectasis. In the BSI, while most of the other clinical and laboratory features score at least 2, the extent of bronchiectasis, as defined by 3 lobes or more, only warrants a score of 1. As such, we believe that HRQOL in bronchiectasis could be similar in patients with bronchiectasis involving fewer than three lobes versus those with three or more lobes.

We also evaluated the responsiveness of the TC‐BHQ and observed a statistically significant improvement in the total score between baseline and follow‐up. The effect size was classified as “small to moderate” (Cohen’s *d* = 0.35). This small degree of improvement is expected, as all patients were followed up in a specialist clinic, where consistent management and optimized care may have contributed to gradual, rather than dramatic, changes in scores over time.

The internal consistency of the TC‐BHQ, evaluated using Cronbach’s alpha, was marginally below the commonly accepted standard (*α* = 0.68 compared to the acceptable threshold of 0.7). This lower alpha value may indicate a lack of homogeneity among the TC‐BHQ items or suggest the need for refinement to more accurately encapsulate the intended construct. The item analysis found that Item 4 (“My chest has felt clear”) demonstrated poor correlation with the total score (corrected item‐to‐total correlation = 0.09), likely attributable to its positive wording contrasting with the other negatively worded items [30].

The test–retest reliability of the TC‐BHQ was found to be acceptable, as demonstrated by an ICC of 0.73. An ICC value between 0.70 and 0.90 generally indicates good reliability, suggesting that the TC‐BHQ provides consistent results when administered to the same individuals under similar conditions over time. This level of reliability is particularly important for a tool designed to monitor longitudinal changes in HRQOL. Since bronchiectasis is a chronic condition that can fluctuate in severity, a reliable measure is crucial to accurately track changes in a patient’s HRQOL over time.

The acceptable 1CC of 0.73 indicates that the TC‐BHQ is sufficiently stable to be used in both clinical and research settings, where repeated assessments are often needed to evaluate the impact of interventions, disease progression, or recovery. Moreover, the test–retest reliability supports the practical utility of the TC‐BHQ in longitudinal studies. It ensures that observed changes in a patient’s quality of life are likely due to real changes in their condition rather than inconsistencies in the measurement tool itself. This reliability is essential for clinicians and researchers aiming to monitor treatment efficacy or disease outcomes over time.

While a simplified Chinese/Mandarin version is available [19], our TC‐BHQ has a significant difference with the simplified Chinese/Mandarin version, given the intrinsic difference in the grammar and wordings in simplified Chinese and traditional Chinese. TC‐BHQ is more appropriate for people living in places that use traditional Chinese.

### 4.1. Limitations and Future Research

The limitations of this study include the modest sample size and the short interval for test–retest reliability assessment, which may have affected the stability of the scores. Larger longitudinal studies may also provide a more comprehensive understanding of the TC‐BHQ’s sensitivity to change over time and its ability to reliably measure treatment effects.

## 5. Conclusion

The TC‐BHQ is a promising instrument for assessing HRQOL in non‐CF bronchiectasis patients. While the reliability measures suggest room for improvement, the TC‐BHQ has the potential to be a useful tool in both clinical settings and research once it is further refined.

## Author Contributions

All authors made a significant contribution to the work reported, whether that is in the conception, study design, execution, acquisition of data, analysis and interpretation, or in all these areas, and took part in drafting, revising, or critically reviewing the article.

## Funding

The study was supported by the Hong Kong Lung Foundation Research Grant.

## Disclosure

All authors gave final approval of the version to be published, have agreed on the journal to which the article has been submitted, and agree to be accountable for all aspects of the work.

## Ethics Statement

This study was performed in accordance with the Declaration of Helsinki. This human study was approved by the Institutional Review Board of the University of Hong Kong and Hospital Authority Hong Kong West Cluster (approval: UW 22‐656 and UW 22‐317).

## Consent

All adult participants provided written informed consent to participate in this study.

## Conflicts of Interest

The authors declare no conflicts of interest.

## Data Availability

All available data are presented in the manuscript, and no additional data will be provided. Data are not available to be shared.
